# Intravenous Immunoglobulins: Mode of Action and Indications in Autoimmune and Inflammatory Dermatoses

**DOI:** 10.1155/2016/3523057

**Published:** 2016-01-18

**Authors:** Lyubomir A. Dourmishev, Dimitrina V. Guleva, Ljubka G. Miteva

**Affiliations:** Department of Dermatology and Venereology, Medical University-Sofia, Bulgaria

## Abstract

Intravenous immunoglobulins (IVIGs), a mixture of variable amounts of proteins (albumin, IgG, IgM, IgA, and IgE antibodies), as well as salt, sugar, solvents, and detergents, are successfully used to treat a variety of dermatological disorders. For decades, IVIGs have been administered for treatment of infectious diseases and immune deficiencies, since they contain natural antibodies that represent a first-line defense against pathogens. Today their indication has expanded, including the off-label therapy for a variety of autoimmune and inflammatory diseases. In dermatology, IVIGs are administered for treatment of different disorders at different therapeutic regimens, mostly with higher doses then those administered for treatment of infectious diseases. The aim of this prospective review is to highlight the indications, effectiveness, side effects, and perspectives of the systemic treatment with IVIGs for patients with severe, life-threatening, and resistant to conventional therapies autoimmune or inflammatory dermatoses.

## 1. Introduction

Intravenous immunoglobulins (IVIGs) have been initially used to treat primary and secondary immune deficiencies, since they contain natural antibodies (Ab) that are first-line defense against pathogens. However over the past decades their indications have expanded tremendously, including the off-label therapy for a variety of autoimmune and inflammatory diseases in dermatology.

IVIG consists of mainly IgG (IgG_3_, IgG_4_) antibodies as well as variable amounts of proteins; IgA, IgE, and IgM Ab; albumin; salt and sugar content; solvents and detergents, depending of the methods of commercial preparation. There are different preparations of IVIG in the market for intravenous administration in two forms—lyophilized and liquid. The first form has to be diluted with water, saline, or 5% glucose while the liquid form (0.5% or 10% solution) is ready to use.

The mechanism of action of IVIG in most autoimmune diseases remains unclear [1]; however various mechanisms have been proposed. IVIGs have an immunomodulatory activity based on biological processes that are implicated in innate or acquired immune response (Table 1).

## 2. Material and Methods

We reviewed prospective clinical studies on the effectiveness of IVIG for treatment of various allergic, autoimmune, inflammatory, and drug induced dermatoses. A standardized literature search was performed using MEDLINE database and the criteria were limited to case reports, clinical studies, and abstracts. Several indications are still controversial due to the lack of controlled clinical research results.

## 3. Results and Discussion

Although in second line, IVIGs have shown promising results in treatment of various autoimmune and inflammatory dermatoses.

## 4. Adverse Drug Reactions

The most significant and potentially life-threatening disorders from the adverse drug reactions group are* Stevens-Johnson syndrome (SJS)* and* toxic epidermal necrolysis (TEN)*. SJS/TEN are adverse skin drug reactions that typically involve the skin and the mucous membranes representing the same disease at different levels of severity. These diseases are characterized by the rapid onset of keratinocyte cell death resulting in the separation of the epidermis from the dermis. The difference between SJS and TEN relates to the body surface affected. SJS consists of less than 10% of the body surface detachment; between 10% and 30% is SJS/TEN overlap syndrome, and if more than 30% of the body surface is affected, the diagnosis is TEN [16]. Moreover it was found that IVIG perfusions bring a significant increase in IgG concentration in the serum, blister fluid, and epidermis of both TEN-involved and clinically uninvolved skin. This highlights the direct effect of infused IgG in the local areas in addition to being present in general circulation, since IgA and IgM remain unchanged [17]. There is still no consensus about the treatment of both disorders, since SJS tend to be distinguished from TEN not only in clinical affection, but in therapeutic responses to corticosteroids and IVIG [18]. Numerous case reports have analyzed the therapeutic effect of IVIG in TEN using total doses greater than 2 g/kg over 3-4 days [13, 19–21], suggesting that total doses of 2 g/kg or less are more likely insufficient to obtain optimal therapeutic effect [22]. In contrast, even low doses IVIG (0.4 g/kg per day) administrated for 5 consecutive days are found highly effective in 87.5% of SJS patients [23]. Hence IVIG together with the systemic steroids should be considered as an important treatment modality for patients with refractory SJS/TEN [23].

## 5. Autoimmune Bullous Diseases

Pemphigus is a group of autoimmune bullous diseases caused by circulating Ab against adhesion molecules located on the keratinocyte surface. In* pemphigus vulgaris*, the Ab are directed against desmoglein-3 and/or desmoglein-1 while in* pemphigus foliaceus *they are directed only against desmoglein-1. High doses of corticosteroids and immunosuppressive drugs are used as first-line therapy in autoimmune bullous dermatoses. In some cases patients do not respond or tolerate high doses of these drugs or the tapering of the steroids can cause new disease flare-ups or in other cases the therapy could lead to side effects. IVIG is an alternative treatment. It is suggested that IVIG decreases serum level of pemphigus Ab by increased catabolism or by manipulating the idiotypic network [24]. Another suggestion is that IVIG can inhibit the binding of antidesmoglein-3 Ab to recombinant desmoglein-3 in a dose dependent manner in vivo and in vitro. In 2002 Bystryn et al. [25] and in 2006 Baum et al. [26] reported 6 and 12 cases of therapy-resistant patients with pemphigus vulgaris which have been treated with IVIGs with a rapid improvement of the diseases activity. The therapy scheme protocol proposed 2 g/kg over 3–5 days (1 cycle) every month with minor side effects. The treatment with IVIGs in* pemphigus foliaceus* has shown also very good response with long remission after the discontinuation of the study drug [27].

*Bullous pemphigoid,* a subepidermal blistering disease, is characterized by the presence of IgG Ab against hemidesmosomal antigens BP230 (BPAg1) and BP180 (BPAg2). There are reported cases with a positive response of BP to IVIG with dose of 2 g/kg per month cycle over 3 months or initially as an adjunctive therapy [28, 29].

*Pemphigoid gestationis* (PG), an autoimmune blistering disease in pregnancy, is characterized by the Ab against BP antigens in the basement membrane zone (BMZ). Although a second-line treatment, IVIG (2 g/kg/cycle, every two weeks antepartum and every three weeks for three months postpartum) was successfully used in a case with clinical and immunological cure, healthy neonate, and lack of adverse events [30].

*Cicatricial pemphigoid* is a form of pemphigoid, affecting the mucous membranes. IVIGs have been given at 2 g/kg/cycle initially every 2-3 weeks as an alternative option to suppress the disease progression [31, 32].

*Epidermolysis bullosa acquisita* (EBA), an autoimmune subepidermal blistering disease of the skin and mucus membranes, is characterized by the presence of IgG Ab (in most patients) targeting the noncollagenous (NC1) domain of type VII collagen, the major component of anchoring fibrils that connect the basement membrane to dermal structures. The disease is often difficult to treat. There are various cases of a possible benefit of IVIG usually in association with previously introduced immunosuppressive therapy [33, 34].

*Linear IgA dermatosis* is an autoimmune subepidermal vesiculobullous disease characterized by the linear deposition of IgA at the BMZ. It is suggested the IVIGs may be useful in not responding to the conventional therapy patients [35, 36].

## 6. Connective Tissue Diseases

*Dermatomyositis* is an autoimmune disease that affects the skin and muscle as a consequence of a complement mediated microangiopathy and T cell mediated muscle destruction. The first-line treatment is high doses of systemic steroids as a monotherapy or combined with immunosuppressive drugs (azathioprine, methotrexate, and cyclosporine) but the side effects are common. It is believed that IVIG could limit the migration of activated T cells from capillaries towards the muscle fibers. Dalakas et al. [37] have conducted a controlled trial of high doses of IVIGs. They found that IVIG at a dose of 2 mg/kg per month was proven to be highly effective in improving both skin and muscle involvement. Other regimens include IVIG 0.1 g/kg/day for 5 days/week for 2 weeks [38] or 0.4 g/kg/day for 5 consecutive days [39]. The response is observed within 3 to 4 months in about 80% of the treated patients. Those patients who do not respond to the first-line therapy should receive IVIG in association with corticosteroids. Similar results, showing an improvement of both muscle and skin disease, have been reported in various forms of inflammatory myopathies as* polymyositis*,* juvenile dermatomyositis* [40], or* necrotizing myopathy* [41]. Moreover IVIG treatment affects well the dermatomyositis complications as ulcerative skin lesions and* calcinosis cutis* [42].

Although first results were not encouraging, IVIGs now present excellent efficacy in steroid resistant cases of* subacute cutaneous lupus erythematosus* and* systemic lupus erythematosus* in a regimen of two-day course (1 g/kg/24 h) every 4 weeks [43]. Other regimens include IVIG 1 g/kg/24 h on 2 consecutive days, followed by 0.4 mg/kg per month for 6 months, or 0.5 g/kg/24 h on 4 consecutive days (2 g/kg/month) for 3 months [44].

*Systemic sclerosis (SSc)*, other connective tissue disease, is characterized with cutaneous fibrosis and internal organ affection. IVIG administered at 2 g/kg over 5 days monthly for 6 months was found to reduce the disease activity in an open label study [45]. Other case reports also show improvements in skin scores suggesting that IVIG could be of benefit to treating SSc's skin involvement. Same results have been reported for treatment of* mixed connective tissue disease* with or without myositis overlap [46].

## 7. Allergic Diseases

*Atopic dermatitis*, a chronic inflammatory skin diseases characterized by a dysregulation of the immune response, usually starts in early infancy but an adult-onset variant is also recognized. Data in literature showed significant children improvement by monotherapy IVIG, given as 2 g/kg [47], but in adults it does not appear to have the same efficacy [48].

*Chronic urticaria* is a skin disorder characterized by recurrent transitory itchy wheals affecting patients for 6 weeks or longer. In the largest study in chronic urticaria, 2 g/kg IVIG has been used over 5 days. Most patients responded clinically with complete and prolonged remission [49].

## 8. Vasculitis and Vasculopathies

*Idiopathic thrombocytopenic purpura (ITP)* is an isolated thrombocytopenia with normal bone marrow and in the absence of other causes of thrombocytopenia presents clinically with petechiae or purpura on skin and signs of mucosal bleeding (gastrointestinal, menometrorrhagia, and haematuria). Therapeutic regimens depend on the patient age (childhood or adults) and clinical course (early diagnosed or chronic) as well as the economical concerns. The standard scheme in childhood ITP is IVIG at a total dose of 0.8–1.0 g/kg given on 1 or 2 consecutive days [50]; however excellent results with very low doses of IVIG (0.1–0.2 g/kg) have also been reported [51]. In adults with ITP, the leukopenia or the serum anti-GPIb-IX Ab are signs for poor IVIG treatment response [52].

*Kawasaki disease* (KD) is an acute febrile vasculitic syndrome of early childhood, affecting predominantly medium-sized blood vessels and particularly the coronary arteries. Skin and mucous membrane lesions include maculopapular or target-like exanthema, followed by desquamation, pharyngitis, and conjunctivitis. In a trial of 549 children with KD, Newburger et al. [53] have shown that a single dose IVIG 2 g/kg in addition to acetylsalicylate is more effective than the conventional regimen of acetylsalicylate and 4 days of IVIG 0.4 g/kg/day.

*Pyoderma gangrenosum (PG)* is uncommon, ulcerative cutaneous disorder of uncertain etiology, claimed by some authors to be as Wegener's arteritis—a type of segmental ulcerative vasculitis [54]. It is associated with systemic diseases in at least 50% of patients [55]. Immunosuppressors are the first line of treatment but the therapeutic effects are variable. Significant improvement, long-lasting remissions, or complete healing is reported in all observed patients with PG treated with 2 g/kg/cycle IVIG [56–58]. Thus IVIG is an important therapeutic option in pyoderma gangrenosum refractory to standard immunomodulatory treatment.

## 9. Other Noninfectious Cutaneous Disorders

*Scleromyxoedema* is a rare skin disorder characterized by fibroblast proliferation and mucin deposition in the dermis of unclear etiology. Lesions can be localized on the skin of the head, neck, and dorsum of the hands but in some cases there is extracutaneous manifestation in heart, lung, joints, and oesophagus. The treatment is difficult and often ineffective. Many therapeutic approaches have been tried. Improvement in skin and systemic involvement of the disease was observed in patients treated with 2 g/kg IVIG over 5 days [59, 60].

*Pretibial myxoedema* (PTM) is characterized by localized lesions of the skin resulting from the deposition of hyaluronic acid, usually as a component of thyroid disease. Although the condition is most often confined to the pretibial area, it may occur anywhere on the skin and it is typically associated with Grave's disease. All patients with localized myxoedema have high serum concentrations of thyroid-stimulating hormone receptor Ab, indicating the severity of the autoimmune condition. Current treatment modalities are at the most cases palliative. There are two studies' data of the use of IVIG published in literature. The largest one [61] showed clinical improvement of the skin lesions, ophthalmopathy, and decreasing of Ab levels with a dose of 2 g/kg in 3 weekly cycles for a total of 7–15 cycles of IVIG compared with a group of patients treated with systemic steroids as monotherapy without improvement. Terheyden et al. [62] reported a group of patients with an elephantiasic form of PTM with no response to 2 g/kg IVIG after 6 monthly cycles but there is a reduction in anti-TSH receptor antibody levels.

## 10. Interactions, Contraindications, and Adverse Effects

Before the initiation of IVIG infusion, it is important to test liver and renal function and complete blood cell count, as well as to perform viral hepatitis screening. Vital signs should be monitored before the infusion, every 15 minutes for the first hour of infusion, and every 30 minutes for the rest of the infusion. The infusion should be at a slow rate and then increasing every 15 minutes. There are no known drug interactions; however IVIG should not be applied at the time of attenuated live vaccinations or within the 3-month period after vaccine.

The therapeutic usage of IVIGs is associated in most cases with a low incidence of side effects according to WHO criteria. IVIGs are basically considered as a safe and efficacious therapeutic option but it is still associated with some adverse effects. They are divided into two groups—minor and severe side effects [63]. Side effects in the first group are transient and often appear during the infusion or up to 72 h following the infusion. It is characterized by headache, nausea, fever, vomiting, cough, malaise, muscle, join and abdominal pain, flushing, urticarial lesions, and variations in heart rate and blood pressure. These reactions are probably due to aggregated immunoglobulin molecules that cause the complement system activation, antigen-antibody, contaminants, or stabilizers reactions [64]. Premedication with systemic steroids, antihistamines (diphenhydramine 50 mg), and nonsteroidal anti-inflammatory drugs (acetaminophen 650 mg) can minimize or prevent them [63]. Leukopenia, neutropenia, and monocytopenia are also seen in IVIGs therapy; however in most of cases those side effects appear to be self-limited and do not lead to increased susceptibility to infection.

Adverse effects in the second group are rare but severe. Most of them appear during or after the first infusion; hence it is important to have a complete medical history before the administration of IVIG. Some of these group side effects are aseptic meningitis, acute renal failure, stroke, exacerbation of the preexisting congestive heart failure, infections, hemolysis, deep venous thrombosis, pulmonary embolism, and anaphylactic shock [26, 63, 64]. Skin adverse effects include severe dermatitis resembling pompholyx initially localized to the palms or soles that then extends to the rest of the body [65], suggesting that a rapid increase in serum IgG levels may be involved in the development of skin lesions [66].

## 11. Conclusion

The number of first-line or high- or medium-priority indications for IVIG treatment in hematology, neurology, rheumatology, intensive care, and dermatology continues to extend. At present,the use of IVIGs is an important step in the treatment of autoimmune dermatoses. As was already stated, they are safe, well-tolerated, and well-accepted therapeutic modality. Reports in literature about the benefits of IVIG are increasing although we still need evidence-based data to improve the safety and efficacy of the drug, however, and supporting data in many indications.

## Figures and Tables

**Table 1:** Mechanism of action of IVIG in inflammatory and autoimmune dermatoses.

Biologic target	Mode of action	Reference
T-lymphocytes	Inhibition of T cell derived IL-2, IL-10, TNF-*β*, and IFN-*γ* Antibodies against CD4 cells, HLA class I and II molecules, and T cell receptor *β* chain	[2–4]

B-lymphocytes	Antibody neutralization and inhibition of Ab production due B-lymphocytes binding Inhibition of IL-6 and TNF-*α* production Induction of B-cell apoptosis	[5–7]

Monocyte/macrophage system	Suppressing the phagocytosis of antibody-coated cells by Fc blockade Anti-inflammatory activity of IVIG through the surface expression of inhibitory Fc *γ*RIIB receptor IVIG increasing the synthesis of IL-8 in cultures of monocytes	[8] [9] [2, 10]

Dendritic cells	Suppression of the dendritic cells differentiation maturation and capacity to secrete IL-12 on activation	[11, 12]

Keratinocytes	Blockage of Fas-mediated keratinocyte death by binding to the CD95 (death receptor)	[13, 14]

Complement system	IVIG having immediate and long-lasting attenuating effect on complement amplification *in vivo* by stimulating inactivation of C3 convertase precursors	[15] [16]

## References

[1] Kazatchkine M. D., Kaveri S. V. (2001). Immunomodulation of autoimmune and inflammatory diseases with intravenous immune globulin. *The New England Journal of Medicine*.

[2] Andersson J., Skansen-Saphir U., Sparrelid E., Andersson U. (1996). Intravenous immune globulin affects cytokine production in T lymphocytes and monocytes/macrophages. *Clinical & Experimental Immunology*.

[3] Hurez V., Kaveri S. V., Mouhoub A. (1994). Anti-CD4 activity of normal human immunoglobulin G for therapeutic use. (Intravenous immunoglobulin, IVIg). *Therapeutic Immunology*.

[4] Marchalonis J. J., Kaymaz H., Dedeoglu F., Schluter S. F., Yocum D. E., Edmundson A. B. (1992). Human autoantibodies reactive with synthetic autoantigens from T-cell receptor *β* chain. *Proceedings of the National Academy of Sciences of the United States of America*.

[5] Tha-In T., Bayry J., Metselaar H. J., Kaveri S. V., Kwekkeboom J. (2008). Modulation of the cellular immune system by intravenous immunoglobulin. *Trends in Immunology*.

[6] Toyoda M., Pao A., Petrosian A., Jordan S. C. (2003). Pooled human gammaglobulin modulates surface molecule expression and induces apoptosis in human B cells. *American Journal of Transplantation*.

[7] Kazatchkine M. D., Dietrich G., Hurez V. (1994). V region-mediated selection of autoreactive repertoires by intravenous immunoglobulin (i.v.Ig). *Immunological Reviews*.

[8] Dwyer J. M. (1992). Manipulating the immune system with immuneglobulin. *The New England Journal of Medicine*.

[9] Samuelsson A., Towers T. L., Ravetch J. V. (2001). Anti-inflammatory activity of IVIG mediated through the inhibitory Fc receptor. *Science*.

[10] Ruiz de Souza V., Carreno M.-P., Kaveri S. V. (1995). Selective induction of interleukin-1 receptor antagonist and interleukin-8 in human monocytes by normal polyspecific IgG (intravenous immunoglobulin). *European Journal of Immunology*.

[11] Bayry J., Lacroix-Desmazes S., Carbonneil C. (2003). Inhibition of maturation and function of dendritic cells by intravenous immunoglobulin. *Blood*.

[12] Bayry J., Lacroix-Desmazes S., Delignat S. (2003). Intravenous immunoglobulin abrogates dendritic cell differentiation induced by interferon-*α* present in serum from patients with systemic lupus erythematosus. *Arthritis & Rheumatism*.

[13] Viard I., Wehrli P., Bullani R. (1998). Inhibition of toxic epidermal necrolysis by blockade of CD95 with human intravenous immunoglobulin. *Science*.

[14] Prasad N. K. A., Papoff G., Zeuner A. (1998). Therapeutic preparations of normal polyspecific IgG (IVIg) induce apoptosis in human lymphocytes and monocytes: a novel mechanism of action of IVIg involving the Fas apoptotic pathway. *Journal of Immunology*.

[15] Basta M., Fries L. F., Frank M. M. (1991). High doses of intravenous Ig inhibit in vitro uptake of C4 fragments onto sensitized erythrocytes. *Blood*.

[16] Lutz H. U., Stammler P., Bianchi V. (2004). Intravenously applied IgG stimulates complement attenuation in a complement-dependent autoimmune disease at the amplifying C3 convertase level. *Blood*.

[17] Paquet P., Kaveri S., Jacob E., Pirson J., Quatresooz P., Piérard G. E. (2006). Skin immunoglobulin deposition following intravenous immunoglobulin therapy in toxic epidermal necrolysis. *Experimental Dermatology*.

[18] Schwartz R. A., McDonough P. H., Lee B. W. (2013). Toxic epidermal necrolysis: part I. Introduction, history, classification, clinical features, systemic manifestations, etiology, and immunopathogenesis. *Journal of the American Academy of Dermatology*.

[19] Trent J. T., Kirsner R. S., Romanelli P., Kerdel F. A. (2003). Analysis of intravenous immunoglobulin for the treatment of toxic epidermal necrolysis using SCORTEN: the university of Miami experience. *Archives of Dermatology*.

[20] Tan A. W., Thong B. Y., Yip L. W., Chng H. H., Ng S. K. (2005). High-dose intravenous immunoglobulins in the treatment of toxic epidermal necrolysis: an Asian series. *Journal of Dermatology*.

[21] Tan S.-K., Tay Y.-K. (2012). Profile and pattern of Stevens-Johnson syndrome and toxic epidermal necrolysis in a general hospital in Singapore: treatment outcomes. *Acta Dermato-Venereologica*.

[22] Trent J., Halem M., French L. E., Kerdel F. (2006). Toxic epidermal necrolysis and intravenous immunoglobulin: a review. *Seminars in Cutaneous Medicine and Surgery*.

[23] Aihara M., Kano Y., Fujita H. (2015). Efficacy of additional i.v. immunoglobulin to steroid therapy in Stevens-Johnson syndrome and toxic epidermal necrolysis. *The Journal of Dermatology*.

[24] Shoenfeld Y. (2004). The idiotypic network in autoimmunity: antibodies that bind antibodies that bind antibodies. *Nature Medicine*.

[25] Bystryn J.-C., Jiao D., Natow S. (2002). Treatment of pemphigus with intravenous immunoglobulin. *Journal of the American Academy of Dermatology*.

[26] Baum S., Scope A., Barzilai A., Azizi E., Trau H. (2006). The role of IVIg treatment in severe pemphigus vulgaris. *Journal of the European Academy of Dermatology and Venereology*.

[27] Ahmed A. R., Sami N. (2002). Intravenous immunoglobulin therapy for patients with pemphigus foliaceus unresponsive to conventional therapy. *Journal of the American Academy of Dermatology*.

[28] Beckers R. C. Y., Brand A., Vermeer B. J., Boom B. W. (1995). Adjuvant high-dose intravenous gammaglobulin in the treatment of pemphigus and bullous pemphigoid: experience in six patients. *British Journal of Dermatology*.

[29] Ahmed A. R. (2001). Intravenous immunoglobulin therapy for patients with bullous pemphigoid unresponsive to conventional immunosuppressive treatment. *Journal of the American Academy of Dermatology*.

[30] Nguyen T., Alraqum E., Razzaque Ahmed A. (2015). Positive clinical outcome with IVIg as monotherapy in recurrent pemphigoid gestationis. *International Immunopharmacology*.

[31] Urcelay M. L., McQueen A., Douglas W. S. (1997). Cicatricial pemphigoid treated with intravenous immunoglobulin. *British Journal of Dermatology*.

[32] Sami N., Bhol K. C., Razzaque Ahmed A. (2002). Intravenous immunoglobulin therapy in patients with multiple mucosal involvement in mucous membrane pemphigoid. *Clinical Immunology*.

[33] Meier F., Sonnichsen K., Schaumburg-Lever G., Dopfer R., Rassner G. (1993). Epidermolysis bullosa acquisita: efficacy of high-dose intravenous immunoglobulins. *Journal of the American Academy of Dermatology*.

[34] Gourgiotou K., Exadaktylou D., Aroni K. (2002). Epidermolysis bullosa acquisita: treatment with intravenous immunoglobulins. *Journal of the European Academy of Dermatology and Venereology*.

[35] Khan I. U., Bhol K. C., Ahmed A. R. (1999). Linear IgA bullous dermatosis in a patient with chronic renal failure: response to intravenous immunoglobulin therapy. *Journal of the American Academy of Dermatology*.

[36] Goebeler M., Seitz C., Rose C. (2003). Successful treatment of linear IgA disease with salazosulphapyridine and intravenous immunoglobulins. *British Journal of Dermatology*.

[37] Dalakas M. C., Illa I., Dambrosia J. M. (1993). A controlled trial of high-dose intravenous immune globulin infusions as treatment for dermatomyositis. *The New England Journal of Medicine*.

[38] Sadayama T., Miyagawa S., Shirai T. (1999). Low-dose intravenous immunoglobulin therapy for intractable dermatomyositis skin lesions. *Journal of Dermatology*.

[39] Saito E., Koike T., Hashimoto H. (2008). Efficacy of high-dose intravenous immunoglobulin therapy in Japanese patients with steroid-resistant polymyositis and dermatomyositis. *Modern Rheumatology*.

[40] Imataka G., Arisaka O. (2014). Long-term, high-dose intravenous immunoglobulin therapy in a patient with banker-type juvenile dermatomyositis. *Cell Biochemistry and Biophysics*.

[41] Patil A. K. B., Prabhakar A. T., Sivadasan A., Alexander M., Chacko G. (2015). An unusual case of inflammatory necrotizing myopathy and neuropathy with pipestem capillaries. *Neurology India*.

[42] Danieli M. G., Gambini S., Pettinari L., Logullo F., Veronesi G., Gabrielli A. (2014). Impact of treatment on survival in polymyositis and dermatomyositis. A single-centre long-term follow-up study. *Autoimmunity Reviews*.

[43] Lampropoulos C. E., Hughes G. R. V., D'Cruz D. P. (2007). Intravenous immunoglobulin in the treatment of resistant subacute cutaneous lupus erythematosus: a possible alternative. *Clinical Rheumatology*.

[44] Ky C., Swasdibutra B., Khademi S., Desai S., Laquer V., Grando S. A. (2015). Efficacy of intravenous immunoglobulin monotherapy in patients with cutaneous lupus erythematosus: results of proof-of-concept study. *Dermatology Reports*.

[45] Levy Y., Amital H., Langevitz P. (2004). Intravenous immunoglobulin modulates cutaneous involvement and reduces skin fibrosis in systemic sclerosis: an open-label study. *Arthritis & Rheumatism*.

[46] Matsuda M., Miki J., Oguchi K., Tabata K., Ikeda S.-I. (2003). Fasciitis in mixed connective tissue disease successfully treated with high-dose intravenous immunoglobulin. *Internal Medicine*.

[47] Wakim M., Alazard M., Yajima A., Speights D., Saxon A., Stiehm E. R. (1998). High dose intravenous immunoglobulin in atopic dermatitis and hyper-IgE syndrome. *Annals of Allergy, Asthma and Immunology*.

[48] Paul C., Lahfa M., Bachelez H., Chevret S., Dubertret L. (2002). A randomized controlled evaluator-blinded trial of intravenous immunoglobulin in adults with severe atopic dermatitis. *British Journal of Dermatology*.

[49] O'Donnell B. F., Barr R. M., Kobza Black A. (1998). Intravenous immunoglobulin in autoimmune chronic urticaria. *British Journal of Dermatology*.

[50] Parodi E., Giordano P., Rivetti E. (2014). Efficacy of combined intravenous immunoglobulins and steroids in children with primary immune thrombocytopenia and persistent bleeding symptoms. *Blood Transfusion*.

[51] Lee J. H., Lee K. S. (2006). Efficacy of very low-dose (200 mg/kg/d) with short-term intravenous immunoglobulin G therapy according to individual response of acute immune thrombocytopenic purpura in childhood. *Clinical Pediatric Hematology-Oncology*.

[52] Peng J., Ma S.-H., Liu J. (2014). Association of autoantibody specificity and response to intravenous immunoglobulin G therapy in immune thrombocytopenia: a multicenter cohort study. *Journal of Thrombosis and Haemostasis*.

[53] Newburger J. W., Takahashi M., Beiser A. S. (1991). A single intravenous infusion of gamma globulin as compared with four infusions in the treatment of acute Kawasaki syndrome. *The New England Journal of Medicine*.

[54] Dourmishev A. L., Serafimova D. K., Vassileva S. G., Dourmishev L. A., Schwartz R. A. (2005). Segmental ulcerative vasculitis: a cutaneous manifestation of Takayasu's arteritis. *International Wound Journal*.

[55] DeFilippis E. M., Feldman S. R., Huang W. W. (2014). The genetics of pyoderma gangrenosum and implications for treatment: a systematic review. *British Journal of Dermatology*.

[56] Gupta A. K., Shear N. H., Sauder D. N. (1995). Efficacy of human intravenous immune globulin in pyoderma gangrenosum. *Journal of the American Academy of Dermatology*.

[57] Dobson C. M., Parslew R. A., Evans S. (2003). Superficial granulomatous pyoderma treated with intravenous immunoglobulin. *Journal of the American Academy of Dermatology*.

[58] Cafardi J., Sami N. (2014). Intravenous immunoglobulin as salvage therapy in refractory pyoderma gangrenosum: report of a case and review of the literature. *Case Reports in Dermatology*.

[59] Lister R. K., Jolles S., Whittaker S. (2000). Scleromyxedema: response to high-dose intravenous immunoglobulin (hdIVIg). *Journal of the American Academy of Dermatology*.

[60] Kumar N., Rodriguez M. (2004). Scleromyxedema in a patient with multiple sclerosis and monoclonal gammopathy on interferon beta-1a. *Multiple Sclerosis*.

[61] Antonelli A., Navarranne A., Palla R. (1994). Pretibial myxedema and high-dose intravenous immunoglobulin treatment. *Thyroid*.

[62] Terheyden P., Kahaly G. J., Zillikens D., Bröcker E.-B. (2003). Lack of response of elephantiasic pretibial myxoedema to treatment with high-dose intravenous immunoglobulins. *Clinical and Experimental Dermatology*.

[63] Prins C., Gelfand E. W., French L. E. (2007). Intravenous immunoglobulin: properties, mode of action and practical use in dermatology. *Acta Dermato-Venereologica*.

[64] Palabrica F. R., Kwong S. L., Padua F. R. (2013). Adverse events of intravenous immunoglobulin infusions: a ten-year retrospective study. *Asia Pacific Allergy*.

[65] Vecchietti G., Kerl K., Prins C., Kaya G., Saurat J.-H., French L. E. (2006). Severe eczematous skin reaction after high-dose intravenous immunoglobulin infusion: report of 4 cases and review of the literature. *Archives of Dermatology*.

[66] Kurata M., Horie C., Kano Y., Shiohara T. (2015). Pompholyx as a clinical manifestation suggesting increased serum IgG levels in a patient with drug-induced hypersensitivity syndrome/drug reaction with eosinophilia and systemic symptoms. *British Journal of Dermatology*.

